# A 3D organoid platform that supports liver-stage *P.falciparum* infection can be used to identify intrahepatic antimalarial drugs

**DOI:** 10.1016/j.heliyon.2024.e30740

**Published:** 2024-05-08

**Authors:** Shringar Rao, Shahla Romal, Bram Torenvliet, Johan A. Slotman, Tonnie Huijs, Tokameh Mahmoudi

**Affiliations:** a Department of Biochemistry, Erasmus University Medical Centre, Rotterdam, Zuid Holland, 3015, GD, Netherlands; b Department of Pathology, Erasmus University Medical Centre, Rotterdam, Zuid Holland, 3015, GD, Netherlands; c Optical Imaging Centre, Erasmus University Medical Centre, Zuid Holland, 3015, GD, Netherlands; d TropIQ Health Sciences, Nijmegen, Netherlands; e Department of Urology, Erasmus University Medical Centre, Rotterdam, Zuid Holland, 3015, GD, Netherlands

**Keywords:** Malaria, Liver organoids, Disease modelling, Schizonts, *P.falciparum*, Drug screening, Pyrimethamine

## Abstract

Malaria, a major public health burden, is caused by *Plasmodium* spp parasites that first replicate in the human liver to establish infection before spreading to erythrocytes. Liver-stage malaria research has remained challenging due to the lack of a clinically relevant and scalable *in vitro* model of the human liver. Here, we demonstrate that organoids derived from intrahepatic ductal cells differentiated into a hepatocyte-like fate can support the infection and intrahepatic maturation of *Plasmodium falciparum*. The *P.falciparum* exoerythrocytic forms observed expressed both early and late-stage parasitic proteins and decreased in frequency in response to treatment with both known and putative antimalarial drugs that target intrahepatic *P.falciparum*. The *P.falciparum*-infected human liver organoids thus provide a platform not only for fundamental studies that characterise intrahepatic parasite-host interaction but can also serve as a powerful translational tool in pre-erythrocytic vaccine development and to identify new antimalarial drugs that target the liver stage infection.

## Highlights

• *P.falciparum* sporozoites can infect hepatocyte-like cell-containing 3D liver organoids (HLOs).
• HLOs support the intrahepatic maturation of *P.falciparum* exoerythrocytic forms.
• *P.falciparum*-infected HLOs can serve as a platform to identify liver-stage antimalarial drugs.

## Abbreviations

ARID1A -: AT-rich interactive domain-containing protein 1A
BAF -: BRG/BRM-associated
DM -: Differentiation media
EEFs -: Exoerythrocytic forms
EM -: Expansion media
HLCs -: Hepatocyte-like cells
HLOs -: Hepatocyte-like cell liver organoids
PfHSP70 -: *P.falciparum* Heat Shock Protein 70
PfMSP-1 -: *P.falciparum* merozoite surface protein 1
PYR –: Pyrimethamine
SWI/SNF -: Switch/sucrose non-fermentable
Spzs -: Sporozoites

## 1 Introduction

Despite advances in prevention and treatment, malaria, caused by parasites of the genus *Plasmodium,* continues to be a major global health problem responsible for more than half a million deaths every year [1]. COVID-19-related disruptions increased malaria incidence and mortality, with 247 million people infected and almost 620,000 deaths in 2021 alone [1]. *Plasmodium falciparum*, the most virulent and dominant strain, causes the majority of deaths and severe clinical manifestations like cerebral malaria, anaemia, multi-organ complications or coma [2,3]. *Plasmodium* parasites have complex life cycles that require a human host and the mosquito vector of the *Anopheles* genus [4]. Transmission to humans occurs when infected mosquitos inject *Plasmodium* species sporozoites (Spzs) via their saliva during a blood meal [5]. These Spzs travel to the liver and replicate in hepatocytes to form exoerythrocytic forms (EEFs) before they mature and release merozoites that continue the replication cycle in erythrocytes [5]. The therapeutic targeting of *Plasmodium* parasites during its early liver stage is an attractive prospect because disease progression can be hindered before the clinical presentations of the disease that occur during erythrocytic replication of the parasite. Very few of the approved antimalarial drugs like primaquine and tafenoquine target the liver stages of the malarial life cycle, but their inadequate efficiency in individuals with acute haemolytic anaemia inhibits their widespread use [6]. Moreover, the development of resistance to antimalarial drugs poses a significant threat to achieving sustainable malaria control [7]. Due to the lower parasite numbers in the liver, therapeutically targeting the hepatic stage of malaria infection would apply less selection pressure, thereby inhibiting the development of drug resistance. However, the lack of a clinically relevant and readily available model to study liver-stage malaria has hindered both the characterization of and targeted drug development against the hepatic stages of the malaria parasites [8].

Previous studies of liver-stage malaria have relied on the use of hepatoma cell lines such as HepG2 or HC-04 or the use of rodent malaria parasites in animal models [[9], [10], [11]]. Although these studies provided valuable insights toward the basic understanding of hepatic malaria, the abnormal proliferation and dysregulated gene expression of hepatoma cell lines and the evolutionary distance and differences in infection kinetics between rodent and human malaria species render these models inadequate in their physiological and clinical relevance [12]. Primary human hepatocytes support the hepatic stage of *P. falciparum* life cycles, but are limited in supply, cannot be expanded, readily lose their differentiated phenotype in cell culture, have high donor variability, and, since commercially generated from a small pool of donors, may not represent the genetic diversity of humans [[13], [14], [15]]. Induced pluripotent stem cell-derived – hepatocyte-like cells support *P. falciparum* replication, but demonstrate modest infectivity, immature phenotypes and are genetically unstable due to reprogramming [16]. Therefore, there remains a need for a clinically relevant and scalable model to study the pre-erythrocytic stages of *Plasmodium* replication.

Organoids are self-organising, stem/progenitor cell-derived, *in vitro* 3D-organ models of human origin that recapitulate the functionality of the tissue of origin. Organoids derived from human intrahepatic cholangiocytes have emerged as a powerful primary liver culture system that allows for long-term culture and expansion of bi-potent cells that can be differentiated into either hepatocyte-like cells (HLCs) that produce albumin, secrete bile, store glycogen and have cytochrome CYP3A4 activity [17,18], or into cholangiocyte-like cells [18]. Importantly, these organoids can be expanded and biobanked to yield quantities which facilitate the screening of therapeutic compounds. This platform has been previously utilised as a model to study hepatotropic pathogens like Hepatitis B Virus [17], Hepatitis E Virus [19], SARS-CoV-2 [20] and Hepatitis C Virus [21,22], and has been speculated to have the potential to be used in liver-stage malaria research [12]. In this work, we evaluated the efficacy of these human ductal cell-derived organoids differentiated into hepatocyte-like cells, herein referred to as hepatocyte-like cell liver organoids (HLOs), as a model to study pre-erythrocytic malaria. We demonstrate that the HLOs are permissive to *P.falciparum* infection and that *P.falciparum* EEFs undergo intra-hepatic maturation in HLOs. We also evaluate the efficacy of antimalarial drugs targeting hepatic stages and identify a novel compound that inhibits the liver-stage maturation of the parasite. This work presents a powerful novel tool to study liver-stage malaria, thereby fulfilling the urgent unmet need for a model for intrahepatic *Plasmodium* research [12].

## 2 Materials and methods

### 2.1 Seeding of ductal-derived liver organoids and cryopreservation

The Medical Ethical Council of the Erasmus Medical Centre approved the use of liver biopsies for research purposes (reference number: MEC-2014–060) [17], and informed consent was provided by the donors or by their next of kin. After surgical excision, liver biopsies from healthy donor livers used for transplantation were collected in 5 ml of Belzer UW cold storage solution and stored at 4 °C until processing. LOs were generated using the protocol as previously described [23]. Briefly, liver specimens were washed once with Dulbecco's Modified Eagle Medium (DMEM) (Sigma) supplemented with 1 % foetal calf serum (FCS) and with 0.1 % penicillin/streptomycin (PS) (Sigma), minced with a scalpel, and incubated at 37 °C with the digestion solution (collagenase 2.5 mg/ml in Earle's Balanced Salt Solution [EBSS]) for 30 min, followed by further mincing every 10 min until single cells are recovered. The solution was then passed through a 70 μM strainer in a 50 ml tube (GreinerBio) and washed with 45 ml Advanced DMEM (Gibco) supplemented with 1 % phosphatidylserine (PS), 10 mM 4-(2-hydroxyethyl)-1-piperazineethanesulfonic acid (HEPES) (Gibco), and 1 % GlutaMax (Gibco), henceforth Ad+++. Cells were centrifuged at 200 g for 5′, counted, and 10^3^–15^3^ cells were mixed with a Basement Membrane Extract, type 2 (BME) (Pathclear) in a ratio of 1 part Ad+++ and 2 parts BME, and seeded in 25 μl drops in 48-well suspension plates (GreinerBio), and overlaid with 250 μl of human liver organoid isolation medium consisting of Ad+++ supplemented with 1X B27 supplement without retinoic acid (Gibco), 1X N2 supplement (Gibco), 1.25 mM N-acetyl-l-cysteine (Sigma), 20 % (vol/vol) Rspo-1 conditioned medium (Huch et al., 2013), 1.25 % (vol/vol) Wnt3a conditioned medium [24], 10 mM nicotinamide (Sigma), 10 nM recombinant human (Leu15)-gastrin I (Sigma), 50 ng/ml recombinant human epidermal growth factor (EGF) (Peprotech), 100 ng/ml recombinant human fibroblast growth factor 10 (FGF10) (Peprotech), 25 ng/ml recombinant human hepatocyte growth factor (HGF) (Peprotech), 10 μM forskolin (Sigma), 5 μM A8301 (Tocris), 25 ng/ml Noggin (Peprotech), and 10 μM Y27632 Rho Kinase (ROCK) Inhibitor (Sigma). After 7 days, the drops were overlaid with expansion media (EM; Ad+++ supplemented with 1X B27 supplement without retinoic acid (Gibco), 1X N2 supplement (Gibco), 1.25 mM N-acetyl-l-cysteine (Sigma), 20 % (vol/vol) Rspo-1 conditioned medium, 1.25 % (vol/vol) Wnt3a conditioned medium [24], 10 mM nicotinamide (Sigma), 10 nM recombinant human (Leu15)-gastrin I (Sigma), 50 ng/ml recombinant human EGF (Peprotech), 100 ng/ml recombinant human FGF10 (Peprotech), 25 ng/ml recombinant human HGF (Peprotech), 10 μM forskolin (Sigma), and 5 μM A8301 (Tocris)). EM was changed twice a week, and cultures were split in ratios of 1:4–1:8 every 7–10 days according to organoid density by resuspending in 10 ml cold Ad+++, incubating in ice for 30 min, and centrifuging at 200g for 5’. The pellet was incubated for 10 min in TrypLE Express at room temperature and mechanically disrupted by pipetting. After a further wash in Ad+++, cells were resuspended in BME solution and seeded in 24-well suspension plates and overlaid with EM for further expansion, or in Recover cell culture freezing medium (Invitrogen) in Cryotube™ vials (Thermo Scientific) for cryopreservation. Cryopreserved samples were frozen at −80 °C for 2–3 days in a freezing container and then transferred to the liquid nitrogen for long term storage.

### 2.2 Differentiation of liver organoids into hepatocyte-like cells

Cryopreserved LOs from the liquid nitrogen were transferred on dry ice, thawed at 37 °C in a water bath, resuspended in 10 ml of Ad+++ and centrifuged at 200g for 5’ at room temperature. The pellet was resuspended in a ratio of 1 part Ad+++ and 2 parts BME and seeded in 50 μl drops in 24-well suspension plates and then overlaid with EM. The number of drops made depends on the size of the pellet [23]. EM was changed twice a week, and cultures were split in ratios of 1:4–1:8 every 7–10 days according to organoid density as described above. The optimal density of organoids before differentiation should be 100–200 organoids per well with a size of 100–250 μm diameter per organoid containing 50–200 cells per organoid. 6 days before differentiated organoids need to be used, the EM is removed from the BME drops and the drops are washed three times with Ad+++. The BME drops containing the organoids are then overlaid with differentiation media (DM: Ad+++ supplemented with 1X B27 supplement without retinoic acid, 1X N2 supplement, 1 mM N-acetylcysteine, 10 nM recombinant human [Leu15]-gastrin I, 50 ng/ml recombinant human EGF, 25 ng/ml recombinant human HGF, 0.5 μM A83-01, 10 μM N-[N-(3,5-Difluorophenacetyl)-L-alanyl]-S-phenylglycine *t*-butyl ester (DAPT) (Sigma), 3 μM dexamethasone (Sigma), 25 ng/ml BMP7, and 100 ng/ml recombinant human FGF19 (Peprotech)).

### 2.3 *P.falciparum* sporozoite isolation and traversal assay

PfNF54 sporozoites, derived from a patient who had never left the Netherlands [25], were purchased from TropIQ, The Netherlands. To generate the sporozoites, PfNF54 asexual and sexual blood stages were cultured in a semi-automated culture system [26] and sporozoites were produced by feeding *Anopheles stephensi* mosquitos (Sind-Kasur Nijmegen strain, selected for refractoriness and susceptibility to *P.falciparum* [27]) using standard membrane feeding of cultured gametocytes, as previously described [27]. Salivary glands were hand dissected, collected in Leibovitz medium (Thermo Fisher Scientific) and homogenized in a homemade glass grinder. Sporozoites were counted in a Bürker-Türk counting chamber using phase-contrast microscopy. Sporozoites were stored in Leibovitz medium at a concentration of at least 2.5 × 10^6^ Spzs per ml at room temperature for not more than 5 h before they were used for infection. To assess Spz traversal, 5000 Hepatoma HC-04 cells (Homo sapiens HC-04, MRA-975) were seeded 2 days prior to the experiment into a 384 well plate coated with rat tail collagen. The HC-04 human hepatoma cell line was acquired through the Malaria Research and Reference Reagent Resource Centre (MR4) as part of the Biodefense and Emerging Infections Research Resources Repository (BEI Resources). The isolated Spzs were added to the wells at an MOI of 7500 Spzs/well for 1 h in the presence of 0.5 mg/mL tetramethylrhodamine-labelled dextran. As a negative control, Spzs were added in the presence of 10 μM cytochalasin D. Nuclei were stained with DAPI. Traversed cells were quantified by ImageXpress PICO.

### 2.4 Infection of differentiated liver organoids with *P.falciparum* sporozoites

On the day of infection, DM was removed and the BME drops containing 100–200 organoids per well with a size of 500–1000 μm per organoid. The drops were mechanically disrupted and organoids from 3 wells were resuspended in 10 ml ice-cold Ad+++, incubated for 1 h on ice, and centrifuged at 100g for 10′ at 4 °C to remove the BME. Spzs in Leibovitz media were diluted in DM to yield a concentration of ∼75,000 Spzs per 150 μl final volume that is used for infection of differentiated organoids from one well of a 24-well plate containing 100–200 organoids per well with a size of 100–250 μm diameter per organoid, each organoid containing 50–200 HLCs. Therefore, 75,000 Sporozoites were used to infect 18,750 HLCs (one well containing ∼150 organoids that contain an average of 125 cells each), thus using an infection ratio of 4 Spzs for every one HLC. The pellet in the falcon tube containing the LOs from three wells was then resuspended in 450 μl final volume containing ∼225,000 Spzs (75,000 × 3), plated in a standard 24-well plate for cell culture, centrifuged for 5′ at 600g at room temperature, and then incubated at 37 °C for 3hrs. The same protocol without the addition of the Spzs was followed for mock-infected or uninfected controls. After incubation, the suspension containing organoids from three wells in 450 μl was collected in a 10 ml Falcon tube containing 9.55 ml of cold Ad+++, centrifuged at 100g for 10’ at 4 °C and the supernatant removed. The pellet was washed by repeating the previous step 5 more times to completely remove the Spzs. After the last wash, the pellet was resuspended in a mixture of 84 μl of BME and 42 μl of Ad+++, and three drops of 42 μl each were seeded on a chamber slide (Nunc) (one drop of 42 μl on the chamber slide seeded for differentiated organoids collected from or one well of a 24 well plate containing 100–200 organoids per well with a size of 500–1000 μm per organoid before infection). After the drop had solidified, LOs were overlaid with 300 μl DM per well. In the case of drug treatments, 10 μM PYR (Sigma-Aldrich) or 10 μM K98 [28] diluted in DMSO were added to the DM immediately after seeding in the chamber slides on the day of the infection. DM media was replaced every 3 days.

### 2.5 Immunofluorescence assay

On the days of collection, DM from organoids was removed and the drops were washed with 300 μl dPBS. The drops containing the LOs were fixed with 4 % paraformaldehyde (PFA) in dPBS for 20′ at RT. The fixation step also dissolves the BME and the organoids then attach to the bottom of the chamber slide. The PFA is carefully removed and the attached organoids are washed with dPBS and treated with 300 μl of 0.1 M glycine in dPBS for 10′ at room temperature. Organoids are then permeabilised with 0.5 % TritonX-100 in dPBS for 30′ at room temperature, washed with dPBS, blocked with 2 % BSA in dPBS for 2hrs at room temperature, and then incubated with a primary antibody first for 2 h at RT and then overnight at 4 °C on an orbital shaker. The primary antibodies used were mouse anti-Pf MSP-1 (Absolute antibody; 1:150) and rabbit anti-PfHSP70 (StressMarq; 1:150). Samples were then washed once with dPBS before incubation with secondary antibodies: donkey anti-mouse Alexa Fluor 594 (Invitrogen; 1:1000) and donkey anti-rabbit-Alexa Fluor 488 (Invitrogen; 1:400) for 2 h at room temperature. Samples were then counterstained with Hoechst 33342 (Thermo Fisher Scientific; 1:2000) for 15’ at room temperature, washed thrice with dPBS, and the chamber and rubber were detached from the slide. 10 μl of mounting medium (Dako) was added per well and the slide was sealed with coverslips.

### 2.6 Image acquisition and analysis

Fluorescent images were captured on a Leica Stellaris 5 microscope fitted with a 40 × 0.40 NA HC Plan Apo CS2 lens. Samples were excited using 405,488 and 561 nm diode lasers and emission was filtered between 420 and 470 nm, 500–550 nm and 570–620 nm respectively. Tile scans of multiple whole organoids were recorded using the LAS-X navigator software in 2D as a single plane for quantification of EEFs, or in 3D as multiple Z-slices with an interval of 0.1–1 μm for representative images and movies. Images in 2D were subsequently utilised to quantify infection rates by downstream image analysis as follows. All image analysis was performed using Fiji/ImageJ 1.53V [29], and a homemade ImageJ plugin to measure nearest neighbour distances. Source and plugin available at https://github.com/ErasmusOIC/NearestNeighbourAnalysis. A macro was written to detect EEFs in exported 2D images with channel 1 corresponding to the PfMSP-1 maturation signal, channel 2 to the PfHSP70 infection signal, channel 3 the nuclei Hoechst 33343 signal and channel 4 the Differential interference contrast image, to allow for semi-automated quantifications of EEFs. All steps are clearly defined and described in the EEF quantification macro (provided as a.doc document in supplementary material). Briefly, nuclei segmentation is performed using the StarDist2D plugin [30]. The outline of the organoid is based on segmented nuclei. Within the organoid, the mean PfMSP-1 maturation signal, the mean PfHSP70 infection signal and the standard deviation of the PfHSP70 signal are measured. A threshold is applied to the PfHSP70 channel, where the required intensity is at least 5 standard deviations removed from the mean signal inside the organoid. Detected objects are labelled as potential EEFs and further filtered on size (>10 μm^2^), roundness (>0.825), distance to the closest nuclei centre (<9 μm), distance to the edge of the organoid (<110 μm) and the ratios of PfHSP70 signal at the centre and surrounding the potential EEFs compared to the mean PfHSP70 signal inside the potential EEF. These filters were applied to remove false positives such as debris and auto fluorescent dead cells from our selection. Objects that pass all mentioned filters are labelled as an EEF. EEFs are subsequently manually filtered based on the differential interference contrast images (channel 4) to further eliminate false positives arising from debris. The fold increase of the mean PfMSP-1 signal inside EEFs compared to the mean PfMSP-1 signal inside the organoid is determined and outputted as a measure of maturation. Diameters of detected EEFs, the number of nuclei detected in the image and the number of detected EEFs in the image are calculated in the macro and given as output. A sample size of at least ∼2500 imaged HLC nuclei needs to be imaged to assess the infection rate with a 95 % confidence interval and a margin of error of 0.1 in follow-up experiments. The upper and lower 95 % confidence intervals of infection rate based on Poisson distribution using Gamma distribution were calculated using pois.daly function on R studio. An increase in the sensitivity of measurement of infection rate and a decrease in the margin of the 95 % confidence intervals can be obtained by increasing the sample size by imaging more nuclei to be included in the image analysis. For representative 3D videos, multiple Z-slices with intervals of 0.1–1 μm were captured and visualised using the 3D viewer plugin in Fiji/Image J [31].

### 2.7 Total RNA isolation and quantitative RT-qPCR

RNA extraction was performed starting from 1 to 2 wells of a 24-well plate of organoids. Organoids were collected in 1 ml of Trizol reagent (Sigma) and RNA was extracted according to manufacturer's instructions. Purified RNA was treated with DNase I (Invitrogen) for 15 min according to the manufacturer's instructions. cDNA synthesis was performed starting from 300 to 1000 ng of DNAse-treated RNA using Superscript II Reverse Transcriptase (Invitrogen) kit following manufacturer's protocol for random primer cDNA synthesis. cDNA was diluted in 1:2.5 in nuclease-free water and 4 μl of the diluted product was used for real-time PCR with the following reagents: 5 μl of GoTaq qPCR Master Mix (Promega), and 1 μl of 10 mM primer mix. Technical duplicates were run for each sample. Amplification was performed on the CFX Connect Real-Time PCR Detection System thermocycler (BioRad) using the following thermal program starting with 3 min at 95 °C, followed by 40 cycles of 95 °C for 10 s and 60 °C for 30 s. The specificity of the quantitative reverse transcription PCR (RT-qPCR) products was assessed by melting curve analysis. Primers used for real-time qPCR are listed below.

CYP3A4: Fwd 5′-TGTGCCTGAGAACACCAGAG-3’; Rev 5′-GTGGTGGAAATAGTCCCGTG-3′

NTCP: Fwd 5′-GCTTTCTGCTGGGTTATGTTCTC-3’; Rev 5′-CATCCAGTCTCCATGCTGACA-3′

Albumin: Fwd 5′-GCGACCATGCTTTTCAGCTC-3’; Rev 5′-GTTGCCTTGGGCTTGTGTTT-3′

Cyclophilin A: Fwd 5′- TCATCTGCACTGCCAAGACTG-3’; Rev 5′-CATGCCTTCTTTCACTTTGCC-3′

### 2.8 Viability assay

Mock-infected organoids were seeded in 30 μl BME drops in 48 well plates overlaid with 250 μl of DM in duplicate per condition. Organoids were mock-treated with DMSO, or treated with 10 μM PYR or K98. As a positive control, organoids were also treated with 10 μg/ml of puromycin. 6 days post seeding for the mock and drug-treated organoids and 9 days post seeding for two additional wells of mock-treated organoids, 120 μl of the supernatant was removed and 150 μl of Cell-Titre Glo 3D reagent was added to the wells. Solutions in the wells were homogenized by pipetting. After an incubation of 30 min in the dark, the relative luciferase units were measured using a GloMax 96 microplate luminometer using the GLOMAX software (version 1.9.2) (Promega). Readouts were normalised to protein concentration per well as measured by Bradford's assay.

### 2.9 Statistical analysis

All data are means ± standard deviation (SD) as indicated in the figure legends. Statistical significance was calculated using a two-tailed *t*-test or as indicated in the figure legends. Analyses were performed using Prism version 8.3.0 (GraphPad software).

## 3 Results

### 3.1 Hepatocyte-like cell liver organoids can be infected by *P.falciparum*

The first aim was to assess if LOs are susceptible to infection by *P.falciparum* Spzs. Cryopreserved intrahepatic cholangiocyte organoids were used to generate differentiated HLOs using a previously described protocol [17,18,23] that first allows expansion of the organoids in expansion media (EM), followed by culturing them in a differentiation media (DM) to generate functional hepatocyte-like cells (HLCs) [18] (Fig. 1A). Differentiated HLOs were dissociated from their 3D matrix and cultured with *P.falciparum* Spzs for 3 h at a ratio of 4 Spzs for every one HLC before the Spzs were washed away and organoids were re-seeded in their 3D matrix on chamber slides to allow the organoids to maintain their 3D morphology. (Fig. 1A). Quality control of every batch of freshly isolated *P.falciparum* Spzs (NF54 strain) was conducted using a traversal assay and the Spzs could traverse through 12.5–14 % of HC-04 cells (Fig. S1). We also validated that infection with *P.falciparum* Spzs. does not result in the de-differentiation of the HLCs by measuring the gene expression levels of the mature hepatocyte markers CYP3A4, Albumin and NTCP in the HLOs before and after infection and finding no significant differences between the conditions (Supplementary Fig. 1B). 6 days post-infection, the HLOs were fixed and immunostaining was performed to assess productive infection using antibodies against two parasitic proteins, *P.falciparum* Heat Shock Protein 70 (PfHSP70) and *P.falciparum* merozoite surface protein 1 (PfMSP-1). PfMSP-1 expression indicates the maturation of the EEFs inside the hepatocytes into merozoites, while PfHSP70 is expressed in all intrahepatic parasitic forms. Productive infection in all eight donors was confirmed by staining for PfHSP70 and PfMSP-1 expression (Fig. 1B and C and S1C; Video S1). To further confirm that these EEFs are robust and maturing, we increased the gain of the nuclei channel to be able to detect and visualise parasite DNA in the EEFs (Fig. 1D and S1D). Due to the observed differences in intensity of the staining of the HLC nuclei DNA and the parasite DNA, a high gain for the nuclei channel resulted in oversaturation of the HLC nuclei images but rendered the parasite DNA in the EEFs clearly visible (Fig. 1D and S1D). Therefore, parasite DNA could also be detected in the EEFs along with PfHSP70 and PfMSP-1. EEFs were detected and quantified using a semi-automated image analysis pipeline and the average infection rate was 25.98 (±s.d. 25.68) EEFs per 10^4^ HLCs when using a multiplicity of infection of 4, with high donor-to-donor variability (Fig. 1E). As expected, no EEFs were detected in any mock-infected controls (Fig. 1E). Based on this observed infection rate in this newly characterised model, we can calculate the minimum number of HLCs to be imaged (sample size) per condition to determine the infection rate when using a multiplicity of infection of 4 with a 95 % confidence interval and a margin of error of 0.1 using the following formula “n = (Z^2 * p * (1-p))/(E^2)” where Z is a score of 1.96 corresponding to a confidence interval of 95 %, p is the estimated infection rate of 25.98 EEFs per 10^4^ HLCs and E is the margin of error of 0.1. Using this formula, we determined that we would need a sample size of at least ∼2500 imaged HLCs to assess the infection rate with a 95 % confidence interval and a margin of error of 0.1 in follow-up experiments. Since this is a newly described 3D model for infection, sample size calculation could only be performed retroactively based on the observed infection rate. On average, we imaged >3900 HLCS to acquire the infection rate presented in Fig. 1E, with 7 out of 8 donors meeting sample size requirements to assess the infection rate with a 95 % confidence interval and a margin of error of 0.1 (Supplementary Table 1). The calculated confidence intervals of every individual measurement of infection rate are also depicted in Supplementary Table 1. High donor-to-donor variability was also observed in the sizes of the EEFs, with the average size of all EEFs Day 6 post-infection quantified to be 9.32 (±s.d. 3.17) μm (Fig. 1F). Overall, these results indicated that HLOs are susceptible to *P.falciparum* infection.

**Fig. 1:**
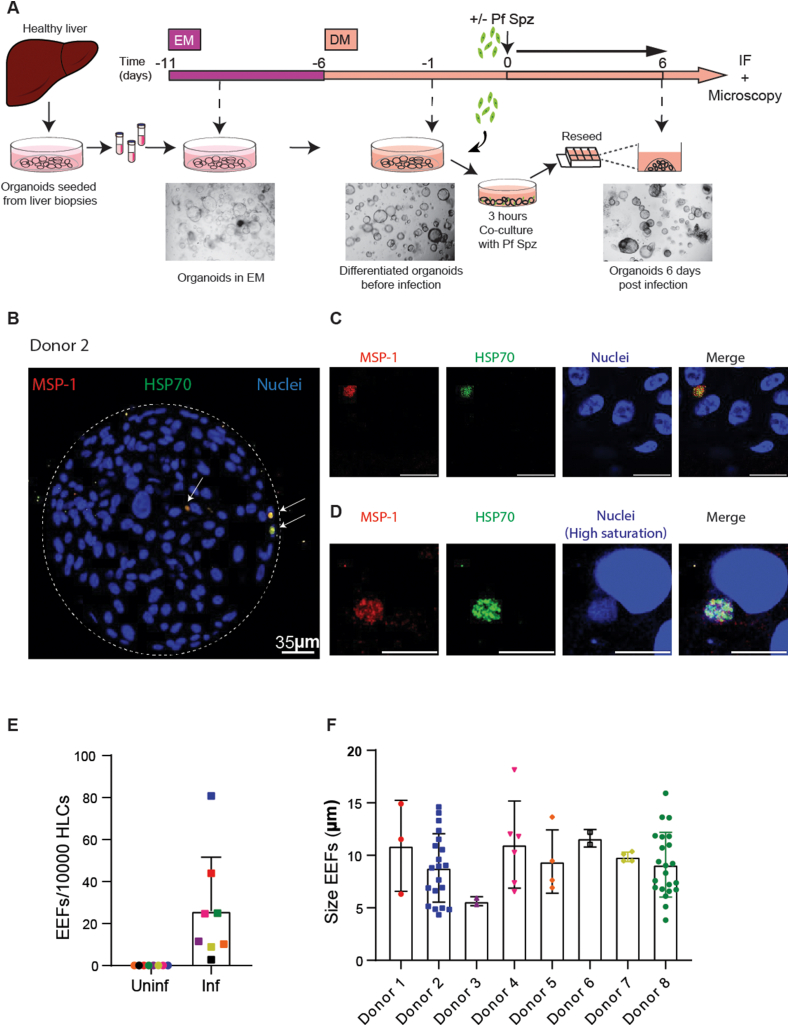
Hepatocyte-like cell liver organoids are susceptible to *P.falciparum* infections (A) Schematic of the protocol for the generation and differentiation of LOs and subsequent infection with *P.falciparum* Sporozoites (Pf Spz). Dotted black arrows point to representative bright-field images of LOs at different stages as indicated. (B) Representative immunofluorescence image of a LO infected with *P.falciparum*. White dotted lines represent the boundaries of the organoid, the white arrows point to EEFs. Red = PfMSP-1, Green = PfHSP70 and Blue = HLC nuclei. Scale bars represent 35 μm (C) Representative immunofluorescence images of EEFs in LOs infected with *P.falciparum*. Red = PfMSP-1, Green = PfHSP70 and Blue = HLC nuclei. Scale bars represent 10 μm (D) Representative immunofluorescence images of EEFs with a high intensity and saturation for the nuclei channel in LOs infected with *P.falciparum*. Red = PfMSP-1, Green = PfHSP70 and Blue = HLC nuclei. Scale bars represent 10 μm (E) Number of EEFs per 10^4^ HLCs. Individual donors are represented by dots of the same colour (n = 8 different biological replicates from independent experiments). Error bars represent mean ± SD. (F) Sizes of EEFs in μm. Each bar represents a different donor (n = 8 different donors) and each dot represents a single EEF. EEFs from the same donor are depicted in the same colour. Error bars represent mean ± SD.

### 3.2 *P.falciparum* EEFs undergo intrahepatic maturation in liver organoids

To characterise EEF maturation, *P.falciparum*-infected HLOs were fixed 3, 6 and 9 days post-infection and stained for PfHSP70 and PfMSP-1 (Fig. 2A). EEFs were observed in HLOs at Days 3, 6 and 9 post-infection (Fig. 2C; Video S2 and S3), while no EEFs were detected in mock-infected controls at any time points (Fig. 2B and data not shown). There was a trend of EEFs increasing in size from Day 3 post-infection compared to later time points, with average EEFs sizes at Day 3, 6 and 9 quantified to be 7.42 (±s.d. 2.02) μm, 9.32 (±s.d. 3.17) μm and 8.4 (±s.d. 3.87) μm respectively (Fig. 2D). The size distributions of the EEF indicated that more EEFs of a larger size were present at Day 6 post-infection than at Day 3 (Fig. 2E). When averaged per donor, the expected donor-specific variability in EEFs sizes was observed, with an increasing trend in EEFs sizes through days with average sizes of 7.72 (±s.d. 2.23) μm, 9.55 (±s.d. 1.88) μm and 9.75 (±s.d. 2.44) μm at Days 3, 6 and 9 post-infection respectively (Fig. S2A). Although differences in infection rates between Days 3, 6 and 9 were not statistically significant, EEF frequency seemed to increase between Day 3 to Day 6 from 9.985 (±s.d. 15.57) at Day 3–32.28 (±s.d. 31.47) EEFs per 10000 HLCs at Day 6 (Supplementary Fig. 2B). Interestingly, a non-significant trend in decrease in EEF frequency on Day 9 compared to Day 6 was also observed, with an infection rate of 19.86 (±s.d. 25.83) EEFs per 10000 HLCs. To validate that this apparent decrease was not due to a reduction in viability of cultures at Day 9 post-seeding, we measured the viability of mock-infected organoids 6 and 9 days post-seeding and observed no differences in viability using Cell-Titre Glo 3D (Supplementary Fig. 2C). The apparent decrease in EEF frequency on Day 9 could be because the merozoites from the EEFs had already egressed. The remaining EEFs detected on Day 9 could possibly have a lag in development and maturation. EEF maturation was quantified by measuring the relative intensity of the PfMSP-1 signal inside the EEF normalised to the intensity of the PfMSP-1 intensity of the organoid. A significant increase in PfMSP-1 expression was observed at Days 6 and 9 post-infection as compared to Day 3 post-infection with the relative PfMSP-1 expression per EEFs being 6.61 (±s.d. 4.8), 12.81 (±s.d. 6.57) and 11.31 (±s.d. 3.85) at Days 3, 6 and 9 post-infection respectively (Fig. 2F). When the PfMSP-1 expression was averaged per donor, a similar increasing trend was observed in PfMSP-1 expression at Days 6 and 9 compared to Day 3 post-infection (Fig. S2D), indicating that the EEFs undergo maturation by Day 6 in HLOs.

**Fig. 2:**
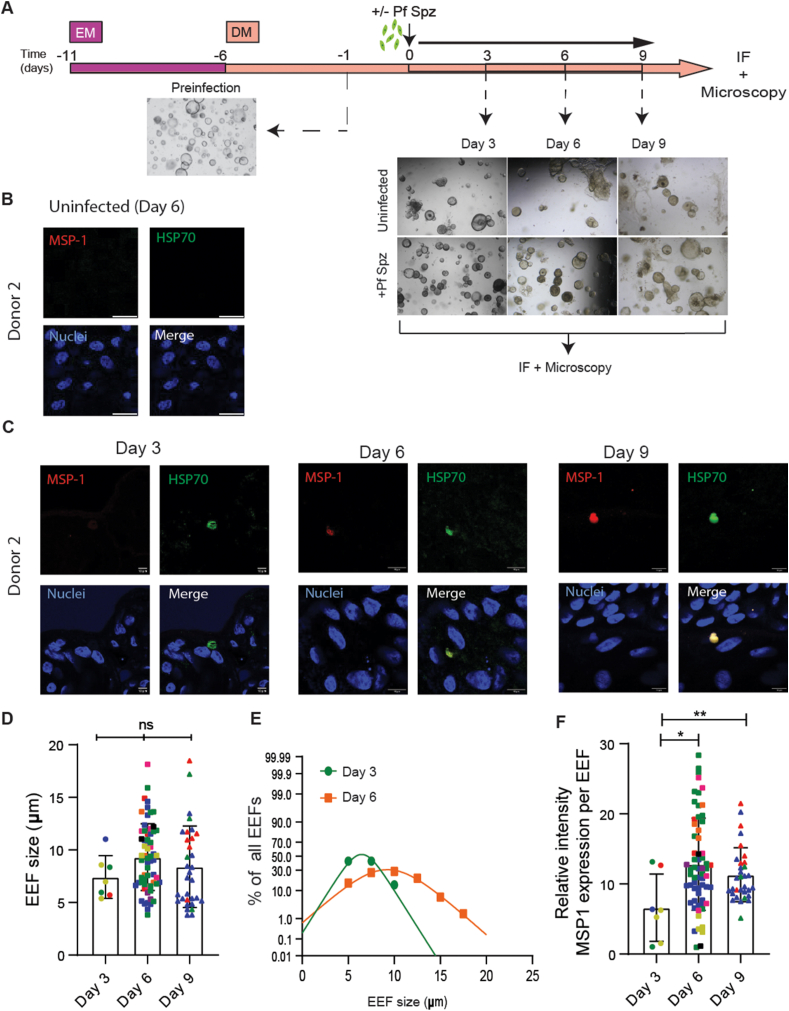
*P.falciparum* EEFs mature in hepatocyte-like cell liver organoids (A) Schematic of the protocol for the differentiation of LOs, subsequent infection with *P.falciparum* Sporozoites (Pf Spz) and collection at different time points. Dotted black arrows point to representative bright-field images of LOs at different time points as indicated. (B) Representative immunofluorescence images of an uninfected LO. Red = PfMSP-1, Green = PfHSP70 and Blue = HLC nuclei. Scale bars represent 10 μm (C) Representative immunofluorescence images of EEFs in LOs infected with *P.falciparum* at three different time points post-infection. Red = PfMSP-1, Green = PfHSP70 and Blue = HLC nuclei. Scale bars represent 10 μm (D) Sizes of EEFs in μm. Data is obtained using five different donors as biological replicates from independent experiments. Each bar represents a different time point post-infection and each dot represents a single EEF detected. EEFs from the same donor are depicted in the same colour. Error bars represent mean ± SD. (unpaired, two-tailed *t*-test, ns: p=>0.05) (E) EEF size distribution from Day 3 (green) and Day 6 (orange) post-infection. (F) Relative expression of the PfMSP-1 signal of the EEFs normalised to the mean PfMSP-1 signal inside the organoid. Data is obtained using five different donors as biological replicates from independent experiments. Each bar represents a different time point post-infection and each dot represents a single EEF detected. EEFs from the same donor are depicted in the same colour. Error bars represent mean ± SD (unpaired, two-tailed *t*-test, *: p=<0.05, **: p=<0.01).

### 3.3 *P.falciparum*-infected liver organoids can be used to assess the efficacy of liver-stage antimalarial drugs

Our next steps were to assess if the *P.falciparum*-infected LO model could be used as a new platform to quantify the efficacy of antimalarial drugs that target the liver stages of parasite development. *P.falciparum*-infected HLOs were reseeded immediately post-infection in culture with DMSO (Mock) or with 10 μM pyrimethamine (PYR), a compound known to inhibit liver-stage development of *P.falciparum* [32] (Fig. 3A). PYR inhibits malaria parasites by repressing the plasmodium dihydrofolate reductase enzyme and interfering with the parasitic folic acid pathway [33]. In addition, PYR is an inhibitor of the BRG/BRM-associated (BAF) complex [34,35], also known as mammalian switch/sucrose non-fermentable (SWI/SNF) complexes. The SWI/SNF complexes also regulate the expression of mammalian dihydrofolate reductase [36]. Therefore, we hypothesised that other inhibitors of the BAF complex could also inhibit parasite development. Therefore, we also treated 5 randomly selected lines of our *P.falciparum*-infected HLOs from Fig. 1E that had sufficient material for downstream analysis immediately post-infection in culture with 10 μM BRD-K98645985 (herein referred to as K98) (Fig. 3A), a small molecule inhibitor of the BAF250a protein (a.k.a. AT-rich interactive domain-containing protein 1A-ARID1A) of the SWI/SNF complex [28]. Treatment with PYR and K98 resulted in a decreased frequency of EEFs from 32.28 (±s.d. 31.47) EEFs/10^4^ HLCs in the Mock treated condition to 7.02 (±s.d. 6.12) and 7.88 (±7.95) EEFs/10^4^ HLCs in the PYR and K98-treated conditions respectively (Fig. 3B). Since sample size calculation was conducted retroactively, one of the donors (in red) presented in Fig. 3B did not meet the sample size requirements of 2500 HLCs. This donor was excluded from the downstream analysis that measures fold change decreases upon drug treatment (Fig. 3C). In the 4 donors that met sample size requirements, a significant decrease in fold change of the frequency of EEFs/10^4^ HLCs compared to the Mock-treated condition was observed upon PYR and K98 drug treatment, with a 67.55 (±s.d. 28.25) and 82.82 (±s.d.15.33) % reduction in EEF frequency respectively (Fig. 3C). Although a reduction in the overall frequency of EEFs was observed, there were no significant differences in the sizes of the remaining EEFs upon drug treatment as compared to the Mock-treated cells (Fig. 3D and S3A). The remaining EEFs showed a decreasing trend in the expression of the PfMSP-1 EEF maturation signal, with a relative expression of 12.16 (±s.d. 6.57), 10.0 (±s.d. 6.30) and 8.73 (±s.d. 5.04) in the Mock, PYR and K98-treated conditions respectively (Fig. 3E). Although when averaged per donor PfMSP-1 expression of the remaining EEFs was not significantly different from the Mock-treated condition (Fig. S2D), the distribution of all EEFs from the Mock, PYR and K98 conditions indicate lower PfMSP-1 expressions upon drug treatment (Fig. 3F). We validated that the effect of these drugs on decreasing parasite infection rate is not due to a loss of viability of the organoids upon drug treatment by measuring the viability of mock-infected organoids treated with PYR and K98 6 days post-infection using Cell-Titre Glo 3D and observing no significant differences on cell viability of the HLOs upon drug treatment (Supplementary Fig. 3C). To assess if the drugs are causing a de-differentiation of the hepatocyte-like phenotype of the HLCs, and subsequently, a reduction in parasite infection rate, we also measured the gene expression levels of mature hepatocyte markers in infected HLOs and HLOs treated with either PYR or K98. As a control, we also measured the gene expression levels of these targets in organoids in expansion media (EM) and as expected found negligible levels of expression of these mature hepatocyte markers in EM organoids (Supplementary Figs. 3D–F). Treatment with PYR did not influence the expression of CYP3A4, Albumin or NTCP levels in HLOs, but treatment in K98 resulted in a significant decrease in gene expression of all three of these targets (Supplementary Figs. 3D–F). To determine if K98 induces reversal of the HLOs to stem-like phenotype, we measured the gene expression level of the stem-cell marker Lgr5 that is highly expressed in organoids in EM and found that K98 does not increase LGR5 expression in HLOs (Supplementary Fig. 3G). These data indicate that although K98 is reducing the parasite infection rate in HLOs, this is most likely a consequence of the HLOs losing the hepatocyte-like phenotype upon drug treatment with K98, in addition to a potential effect as a BAF inhibitor on liver-stage parasites. Overall, these results demonstrate the feasibility of *P.falciparum*-infected HLOs to assess the efficacy of antimalarial drugs that target the hepatic stages of the parasite.

**Fig. 3:**
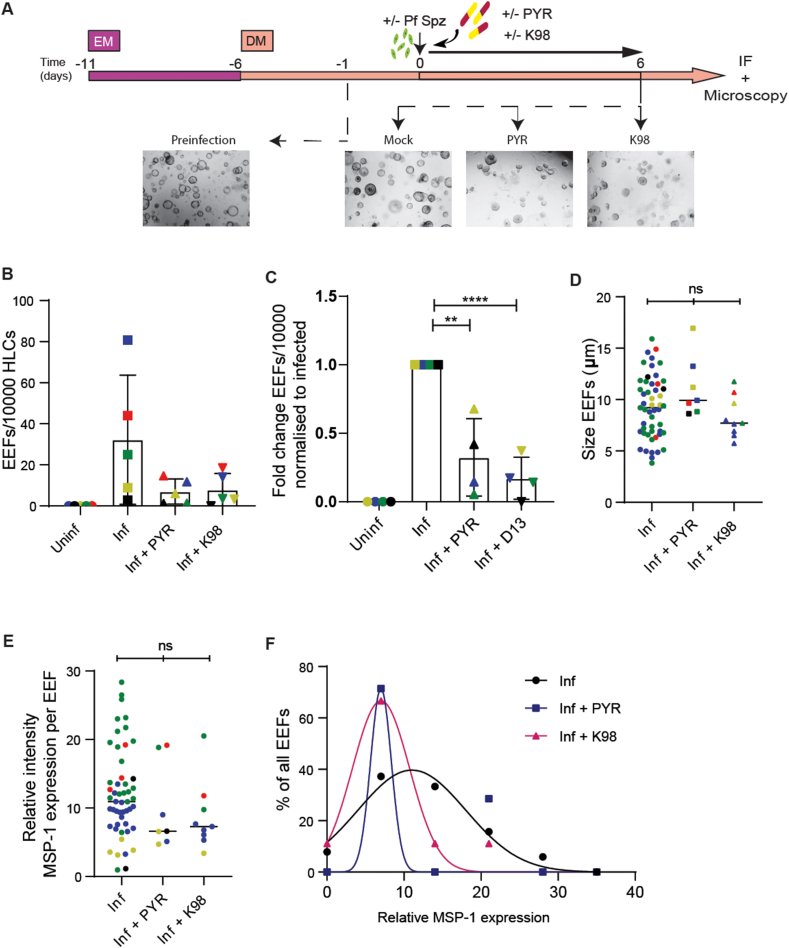
*P.falciparum* EEFs are inhibited by PYR and K98 (A) Schematic of the protocol for the differentiation of LOs, subsequent infection with *P.falciparum* Sporozoites (Pf Spz) and treatment with PYR (10 μM) or K98 (10 μM). Dotted black arrows point to representative bright-field images of LOs at different stages as indicated. (B) Number of EEFs per 10^4^ HLCs. Individual donors are represented by dots of the same colour (n = 5 different donors as biological replicates from independent experiments). Error bars represent mean ± SD. (C) Fold change of number of EEFs per 10^4^ HLCs as compared to the Mock-treated *P.falciparum*-infected condition. Individual donors are represented by dots of the same colour (n = 4 different donors as biological replicates from independent experiments). Error bars represent mean ± SD (unpaired, two-tailed *t*-test, **: p=<0.01, ****: p=<0.0001). (D) Sizes of EEFs in μm. Data is obtained using five different donors as biological replicates from independent experiments. Each dot of the same colour represents a single EEF detected and EEFs observed from the same donor are depicted in the same colour. Error bars represent mean ± SD. (unpaired, two-tailed *t*-test, ns: p=>0.05). (E) Relative PfMSP-1 expression of EEFs normalised to the mean PfMSP-1 signal inside the organoid. Data is obtained using five different donors as biological replicates from independent experiments. Each dot represents a single EEF detected and EEFs from the same donor are depicted in the same colour. Error bars represent mean ± SD. (unpaired, two-tailed *t*-test, ns: p=>0.05). (F) Distribution of relative PfMSP-1 expression of EEFs normalised to the mean PfMSP-1 signal inside the organoid from Mock-treated *P.falciparum*-infected (black), PYR-treated *P.falciparum*-infected (indigo) and K98-treated *P.falciparum*-infected (pink) LOs.

## 4 Discussion

In this study, we demonstrate that intrahepatic cholangiocyte organoids differentiated into hepatocyte-like cells (HLOs) can be infected by *P.falciparum* (Fig. 1) and present a new model to study intra-hepatic stages of the parasite. In HLOs generated from eight different donors, the average rate of infection was ∼26 EEFs/10^4^ HLCs at 6 days post-infection, albeit with high donor-to-donor variability characteristic of primary cell hepatocyte cultures [14,37]. Although this variability can pose a challenge in other primary models due to low infection rates in some donors, the intrahepatic cholangiocyte-derived liver organoid system can be expanded and cryopreserved, allowing for the selection of lines with high *P.falciparum* infection rates for studies, thereby ensuring enough biological material for downstream biological applications to characterise intra-hepatic parasite development. The observed infection rate of ∼0.26 % in our 3D model is comparable to recent primary cell-derived 2D *in vitro* models to study liver-stage malaria that have shown infection rates of ∼0.01 % [16], ∼0.12 % [14] and ∼0.75 % [38]. It may also be possible to increase infection rates by using more Spzs to infect the HLOs and the optimal multiplicity of infection remains to be tested in future studies. The average size of the EEFs observed in HLOs at Day 6 post-infection was 9.32 (±s.d. 3.17) μm (Fig. 2D), comparable to EEF sizes of 10–15 μm as observed in other cell systems at similar time points post-infection [14,16]. Importantly, the EEFs in HLOs also undergo intrahepatic development as demonstrated by the increased expression of the merozoite protein PfMSP-1 at Day 6 post-infection compared to Day 3 post-infection (Fig. 2F).

We present *P-falciparum*-infected HLOs as a new platform to screen for antimalarial drugs that target the liver stage and identify K98 as a small molecule that can inhibit intrahepatic *P.falciparum* development (Fig. 3). We observed that treatment of *P.falciparum*-infected HLOs with pharmacological inhibitors of the BAF complex, PYR and K98, 3 h post-infection could inhibit intra-hepatic parasite development, with a significantly reduced number of EEFs observed upon drug treatment (Fig. 3C). For future drug-screening studies that utilise this model, the sensitivity of the assay can be increased and margins of the 95 % confidence interval can be decreased by increasing the sample size via the acquisition of more images of infected HLCs to be included in the analysis. This is technically feasible using the scale of infection as described in the methods section of this manuscript. Moreover, the remaining EEFs upon drug treatment also had reduced PfMSP-1 expression (Fig. 3E and F), indicating that these drugs could also inhibit intrahepatic schizont maturation. K98 inhibits the ARID1A domain of the BAF complex and we hypothesised that K98 could inhibit parasite development in a mechanism similar to PYR, also an inhibitor of the BAF complex, by interfering with the parasite folic acid pathways. However, K98 also induced the de-differentiation of the HLCs and a decrease in the expression of the mature hepatocyte markers CYP3A4, Albumin and NTCP (Supplementary Figs. 3D–F), and we hypothesize that this is the reason that we observe reduced parasite infection in K98-treated cells. These drugs were administered shortly after infection with sporozoites, indicative of early treatment when compared to a clinical scenario. It would also be interesting to assess the effect of pre-treatment with drugs or treatment 3 days after infection on EEFs development, conditions that would mimic the clinical scenarios of prophylactic treatment or treatment at later stages of the disease. An outstanding question is whether the HLOs could also support the infection of other malaria parasites, especially *P.vivax* which is capable of seeding a reservoir known as hypnozoites in the human liver that is responsible for chronic, relapsing infections weeks to months following primary infection [39], often referred to as the “black box” of malaria research [40]. Differentiated HLOs can be cultured for 1–2 months with intermittent recovery in EM [17], a time frame that is conducive to studying intra-hepatic *P.vivax* hypnozoites.

In this study, we developed a semi-automated image analysis pipeline to allow for unbiased and expedited identification of EEFs in HLOs. This image analysis pipeline to identify EEFs can be further optimised using machine learning techniques to obviate the need for manual verification and facilitate its use in larger-scale drug screen studies. Fluorescent-tagged *P.falciparum* parasites can also be used to infect HLOs to streamline and expedite downstream image analysis. HLOs can also be dissociated into single cells, conducive to the implementation of flow-cytometry-based readouts for infection. HLOs have also been used to assess drug-induced liver injury [17] and it is possible to multiplex experimental readouts to simultaneously assess drug-induced toxicity and anti-malarial activity. The ability of intrahepatic cholangiocyte-derived organoids to be cryopreserved and amplified allows for a steady supply of hepatocyte-like cells that can be seeded in 96 or 384 well plates to conduct medium throughput drug screens to identify novel antimalarial drugs that target the liver.

Apart from use in drug screens, *P-falciparum*-infected HLOs can also be used in studies to discover and characterise host and parasitic factors involved in liver-stage parasite development to identify new targets of therapeutic intervention. Advances in single-cell-omics technologies can be applied to the *Plasmodium*-infected HLOs to investigate intrahepatic parasitic development. HLOs are amenable to genetic modification by CRISPR-Cas or lentiviral-based systems [41,42] allowing for the further characterisation of molecular mechanisms of host factors potentially required for intrahepatic parasite development. The *Plasmodium*-infected liver organoid platform can also be used as a pre-clinical screening platform to assess the efficacy of pre-erythrocytic vaccines for malaria, especially when investigating the efficacy of pre-erythrocytic, live-attenuated sporozoite vaccines by monitoring EEF development in the attenuated strain compared to the wild-type. Recent advances in developing co-culture platforms of organoids with immune cells could further increase the potential of this platform to be utilised in vaccine research [43].

One of the challenges of working with liver organoids is the differences in growth rates between different passages and donors making it harder to standardise organoid sizes, which could potentially cause variability in drug availability and infection rates. Filtering organoids based on their size before seeding can promote standardization in size. In the protocol used in this study [18], the liver organoids are derived from adult resident ductal cells that undergo Tet1-mediated epigenetic reprogramming to assume a stem cell state [44], with some cholangiocyte markers still being expressed upon differentiation into the hepatocyte-like cell fate [21,45]. Modification of the culture media to enhance differentiation into hepatocyte-like phenotypes may result in an increased infection rate. Given the variability in infection rate, -omics analyses on organoid lines with differences in parasite permissiveness could identify host factors contributing to intrahepatic development. The generation of liver organoids requires access to liver tissue and medical ethical approval. Although tissue can be obtained from the material used for liver transplants, needle biopsies can also be used. Patient-derived organoids from people with active infection can be used to study EEF development, with the possibility to correlate to evaluate responsiveness to intrahepatic drugs, especially useful when the donors are infected with parasites that cause relapsing illness. When considering the scalability of HLOs to study malaria, the cryopreservation of liver organoids allows its use in laboratory settings without access to human tissues. It remains to be assessed if the merozoites released from infected HLOs are replication-competent and can further propagate in red blood cells. A limitation of our study is that we were unable to characterise liver to blood stage transition of the parasite. The extremely short time span of 5 min during which time the merozoites have invasive capacity has rendered this virtually impossible to experimentally address at the scale and costs of our currently described *in vitro* model system, a technical challenge that is further accentuated by the 3D nature of our model system. Using advanced microfluidics and bioengineering techniques for our future work, it would be interesting to increase the complexity of our model by adding a vascular component to possibly study the subsequent erythrocytic stages of the *Plasmodium* life cycle. Overall, we present in this report a primary cell-based, 3D organoid system that supports *P.falciparum* infection with the potential for use in fundamental research to identify parasitic or host factors involved in intrahepatic development of the parasite or in drug screens to discover new antimalarial drugs targeting the liver.

## Data availability statement

Data associated with this study is included in the article/supplementary material/referenced in article. Additional microscopy data reported in this paper will be shared by the lead contact upon request. All original code is available in this paper's supplemental information. Any additional information required to reanalyze the data reported in this paper is available from the lead contact upon request. This study did not generate new unique reagents. Further information and requests for resources and reagents should be directed to the lead contact, Dr. Shringar Rao (s.rao@erasmusmc.nl).

## CRediT authorship contribution statement

**Shringar Rao:** Writing – review & editing, Writing – original draft, Validation, Supervision, Methodology, Investigation, Funding acquisition, Formal analysis, Conceptualization. **Shahla Romal:** Writing – review & editing, Methodology, Investigation. **Bram Torenvliet:** Writing – review & editing, Validation, Software, Formal analysis. **Johan A. Slotman:** Writing – review & editing, Software. **Tonnie Huijs:** Writing – review & editing, Resources, Methodology. **Tokameh Mahmoudi:** Writing – review & editing, Supervision, Resources, Methodology, Funding acquisition.

## Declaration of competing interest

The authors declare that they have no known competing financial interests or personal relationships that could have appeared to influence the work reported in this paper.

## Figures and Tables

**figs1:**
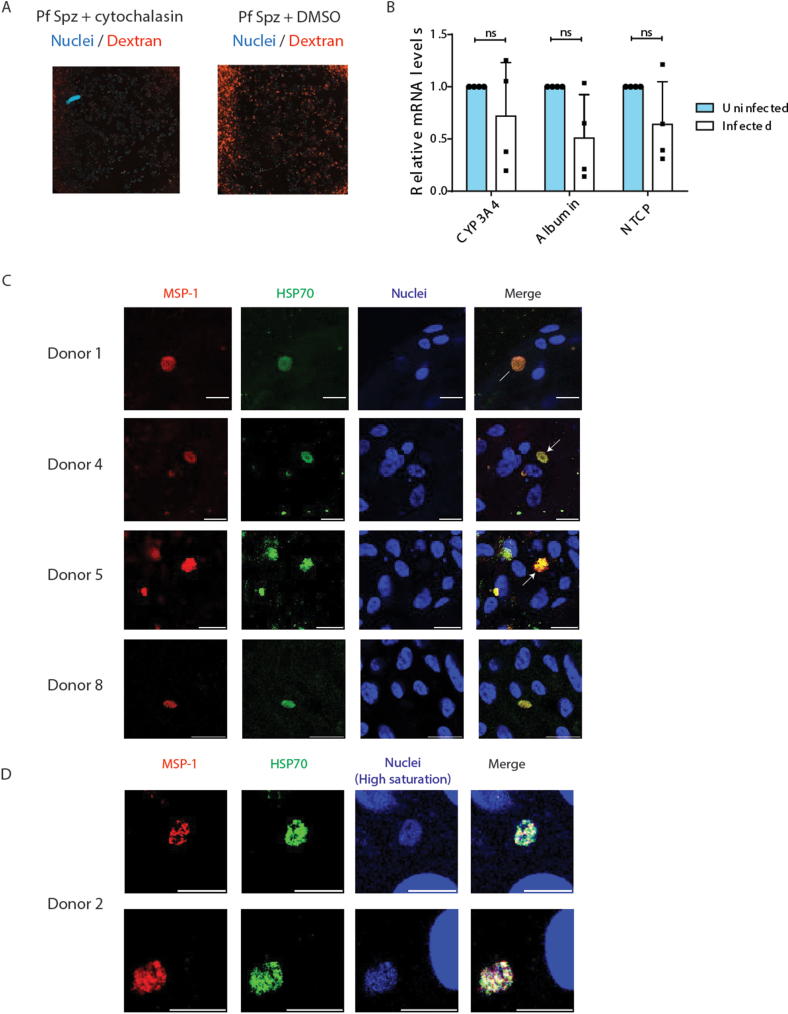
**Figure S1. *P.falciparum* sporozoites infect liver organoids**(A) Representative images of traversal assay of *P.falciparum* Sporozoites in HC-04 cells in the presence of 10 µM cytochalasin (Negative control) or DMSO. Fluorescently labelled dextran indicative of traversal = red, HC-04 nuclei = blue. (B) Relative gene expression of CP3A4, Albumin and NTCP expression levels normalised to housekeeping gene Cyclophillin A. n=4 different donors as biological replicates from independent experiments. (paired, two-tailed t-test, ns: p=>0.05)(C) Representative immunofluorescence images of EEFs in LOs infected with *P.falciparum* from four different donors. Red = PfMSP-1, Green = PfHSP70 and Blue = HLC nuclei. Scale bars represent 10μm. (D) Representative immunofluorescence images of EEFs with a high intensity and saturation for the nuclei channel from one representative donor HLO infected with *P.falciparum*. Red = PfMSP-1, Green = PfHSP70 and Blue = HLC nuclei. Scale bars represent 10μm.

**figs2:**
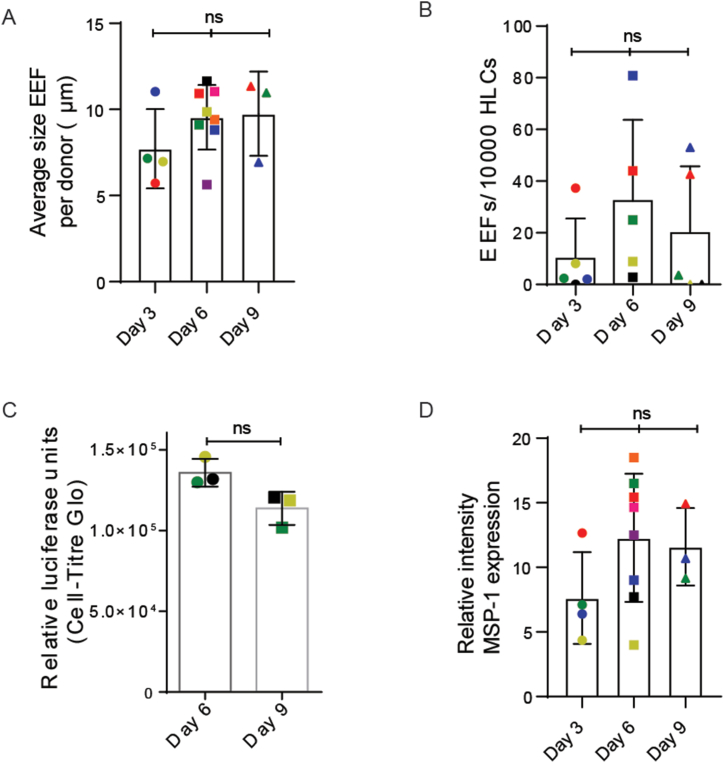
**Figure S2. EEFs morphologies at different time points post *P.falciparum* infection** (A) Average sizes of EEFs in μm per donor. n = at least 3 different donors as biological replicates from independent experiments. Individual donors are represented by dots of the same colour. Error bars represent mean ± SD. (paired, two-tailed t-test, ns: p=>0.05).(B) Number of EEFs per 10^4^ HLCs. n = 5 different donors as biological replicates from independent experiments. Each bar represents a different time point post infection. Individual donors are represented by dots of the same colour. Error bars represent mean ± SD. (paired, two-tailed t-test, ns: p=>0.05).(C) Viability of the HLOs as measured in relative luciferase units. n = 3 different donors as biological replicates from independent experiments. Each bar represents a different time point post infection. Individual donors are represented by dots of the same colour. Error bars represent mean ± SD. (paired, two-tailed t-test, ns: p=>0.05).(D) Average relative PFMSP-1 expression of EEFs per donor. n = at least 3 different donors as biological replicates from independent experiments. Individual donors are represented by dots of the same colour. Error bars represent mean ± SD. (paired, two-tailed t-test, ns: p=>0.05).

**figs3:**
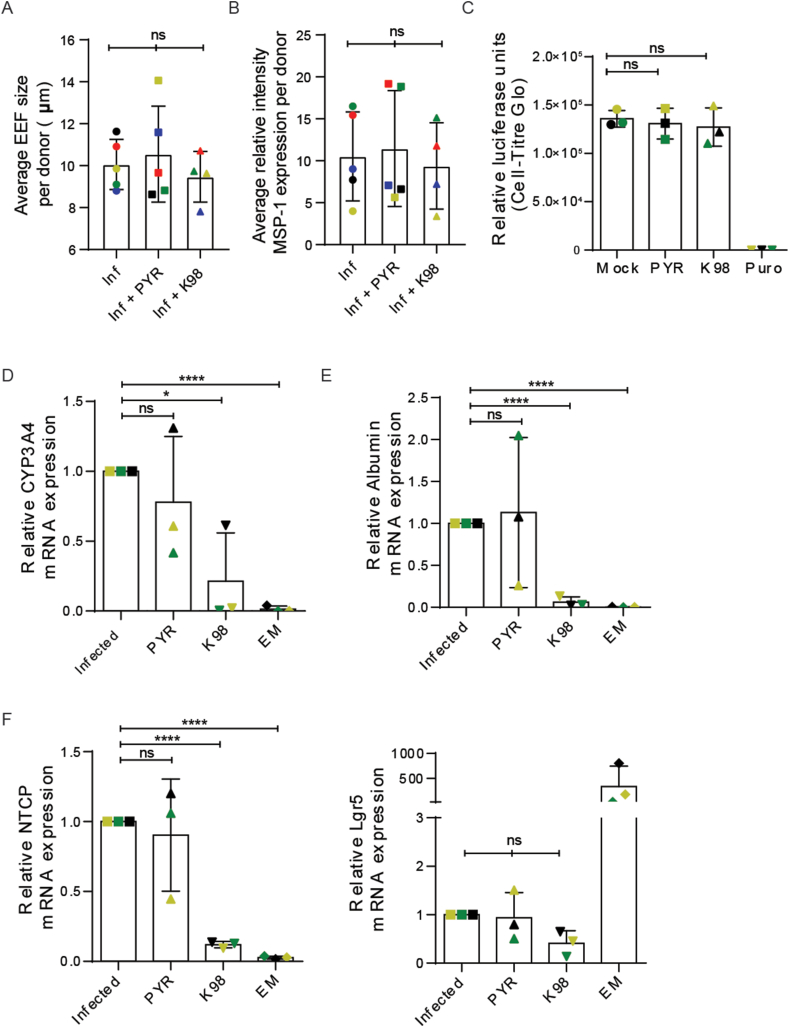
**Figure S3. Effects of antimalarial drugs on EEF size and development**(A) Average sizes of EEFs in μm per donor. n = At least 4 different donors as biological replicates from independent experiments. Individual donors are represented by dots of the same colour. Error bars represent mean ± SD. (paired, two-tailed t-test, ns: p=>0.05).(B) Average relative PFMSP-1 expression of EEFs per donor. n = At least 4 different donors as biological replicates from independent experiments. Individual donors are represented by dots of the same colour. Error bars represent mean ± SD. (paired, two-tailed t-test, ns: p=>0.05).(C) Viability of the HLOs as measured in relative luciferase units. n = 3 different donors as biological replicates from independent experiments. Individual donors are represented by dots of the same colour. Error bars represent mean ± SD. (unpaired, two-tailed t-test, ns: p=>0.05).(D) Relative mRNA levels of CYP3A4 normalised to the housekeeping cyclophillin A. n = 3 different donors as biological replicates from independent experiments. Individual donors are represented by dots of the same colour. Error bars represent mean ± SD. (unpaired, two-tailed t-test, ns: p=>0.05, *: p=<0.05, ****: p=<0.0001).(E) Relative mRNA levels of Albumin normalised to the housekeeping cyclophillin A. n = 3 different donors as biological replicates from independent experiments. Individual donors are represented by dots of the same colour. Error bars represent mean ± SD. (unpaired, two-tailed t-test, ns: p=>0.05, ****: p=<0.0001).(F) Relative mRNA levels of NTCP normalised to the housekeeping cyclophillin A. n = 3 different donors as biological replicates from independent experiments. Individual donors are represented by dots of the same colour. Error bars represent mean ± SD. (unpaired, two-tailed t-test, ns: p=>0.05, ****: p=<0.0001).(G) Relative mRNA levels of LGR5 normalised to the housekeeping cyclophillin A. n = 3 different donors as biological replicates from independent experiments. Individual donors are represented by dots of the same colour. Error bars represent mean ± SD. (unpaired, two-tailed t-test, ns: p=>0.05).

## Appendix A Supplementary data

Raw data Image Analysis Raw data Image Analysis

Image analysis code Image analysis code
