# Supplementary material for: A 3D organoid platform that supports liver-stage P.falciparum infection can be used to identify intrahepatic antimalarial drugs
Source: Heliyon. 2024 May 8;10(10):e30740. doi: 10.1016/j.heliyon.2024.e30740 (PMC11103482; doi:10.1016/j.heliyon.2024.e30740)
Supplement: Image analysis code [file mmc2.docx]

//Requesting user defined variables via dialog

Dialog.create("Selecting parameters for EEF detection.");

Dialog.addNumber("Minimal number of nuclei per organoid:", 5);

Dialog.addNumber("Minimal size of EEF (um^2):", 10);

Dialog.addNumber("Number of standard deviations between mean signal and EEF signal:", 5);

Dialog.addNumber("Maximum distance allowed between EEF and closest nuclei (um):", 9);

Dialog.addNumber("Maximum distance allowed between EEF and organoid boundary (um):", 110);

Dialog.show();

//Selection criteria

min_org_size = Dialog.getNumber();

min_EEF_area = Dialog.getNumber();

fold_from_mean = Dialog.getNumber();

Max_distance_to_nuclei = Dialog.getNumber();

Max_distance_to_edge = Dialog.getNumber();

//Image information

maturation_channel = 1;

EEF_channel = 2;

DAPI_channel = 3;

//Information for blurring and ROI creation

gaussSize_EEF = 5;

gaussSize = 25;

band_size = 10;

R1 = 0.3;

R2 = 3.2;

circ = 0.825;

dir1 = getDirectory("Select the folder containing multiple 2D images");

complete_filename = File.getDirectory(dir1) + File.getName(dir1);

list1 = getFileList(dir1);

save_loc = getDirectory("Select the folder where to save images for quality control");

for(file = 0 ; file<list1.length; file++){

filename = dir1 + list1[file];

open(filename);

getDimensions(width, height, channels, slices, frames);

getPixelSize(unit, pixelWidth, pixelHeight);

origtitle = getTitle();

//Block 1, Finding the organoids & nuclei in the image

//Finding nuclei using StarDist

run("Duplicate...", "title=Dapi duplicate channels="+DAPI_channel);

selectWindow("Dapi");

run("Command From Macro", "command=[de.csbdresden.stardist.StarDist2D], args=['input': 'Dapi', 'modelChoice':'Versatile (fluorescent nuclei)', 'normalizeInput':'true', 'percentileBottom':'0', 'percentileTop':'100', 'probThresh':'0.6500000000000001', 'nmsThresh':'0.4', 'outputType':'Both', 'nTiles':'1', 'excludeBoundary':'2', 'roiPosition':'Automatic', 'verbose':'false', 'showCsbdeepProgress':'false', 'showProbAndDist':'false'], process=[false]");

close("Label Image");

selectWindow("Dapi");

//Excluding false "Nuclei"

run("Set Measurements...", "area redirect=None decimal=0");

roiManager("Deselect");

roiManager("Show all");

roiManager("Measure");

non_Nuclei = newArray();

for(Q=0; Q<(nResults); Q++){

size = getResult("Area", Q);

if(size > 200){//Very large object are likely not nuclei

non_Nuclei = Array.concat(non_Nuclei,Q);

}

}

delete_selection(non_Nuclei);

close("Results");

//Displaying centers of nuclei as points

newImage("Nuclei_Centers", "8-bit black", width, height, 1);

setForegroundColor(255, 255, 255);

Create_points("Nuclei_Centers");

run("Draw", "slice");

//Combine all parts of the organoids, form one ROI

roiManager("Show all");

roiManager("Combine");

roiManager("Delete");

roiManager("Add");

deselect();

//Enlarge the ROI

selectWindow("Dapi");

roiManager("Select", 0);

run("Enlarge...", "enlarge=20");

roiManager("Update");

//In our organoid outline we add a small hole

//This prevents errors during spilling when we only have one organoid

create_hole();

//Splitting into individual organoids

roiManager("Split");

roiManager("Select", 0);

roiManager("Delete");

//Counting the amount of nuclei per ROI and removing small organoids

run("Set Measurements...", "area mean redirect=None decimal=0");

remove_small_organoids(min_org_size);

//Combine all parts of the organoids, form one ROI

roiManager("Show all");

roiManager("Combine");

roiManager("Delete");

roiManager("Add");

deselect();

close("Dapi");

//Count the number of nuclei in the organoid

run("Set Measurements...", "area mean redirect=None decimal=0");

selectWindow("Nuclei_Centers");

roiManager("Show all");

roiManager("Measure");

area = getResult("Area", 0);

mean = getResult("Mean", 0);

number_nuclei = (mean*area)/255;

close("Results");

//Draw the outline of the organoid on new image

newImage("Edge", "8-bit black", width, height, 1);

roiManager("Select", 0);

run("Draw", "slice");

deselect();

//Block 2, Identifying potential EEFs based on intensity properties

//Measuring signal in maturation channel

selectWindow(origtitle);

run("Duplicate...", "title=Maturation duplicate channels="+maturation_channel);

roiManager("Select", 0);

run("Clear Outside");

run("Set Measurements...", "mean redirect=None decimal=0");

roiManager("Measure");

maturation_signal1 = getResult("Mean",0);

close("Results");

//Looking at the intensity histogram of the EEF marker channel in the entire organoid

selectWindow(origtitle);

run("Duplicate...", "title=EEF duplicate channels="+EEF_channel);

run("Gaussian Blur...", "stack sigma="+gaussSize_EEF);

roiManager("Select", 0);

run("Clear Outside");

run("Set Measurements...", "mean standard redirect=None decimal=0");

roiManager("Measure");

average = getResult("Mean",0);

sd = getResult("StdDev",0);

close("Results");

roiManager("Select",0);

roiManager("Delete");

//Thresholding based on its intensity profile

min_int = average+sd*fold_from_mean;

max_int = 255-3*sd;

SD_min = 3*average;

SD_max = 8*average;

setThreshold(min_int, max_int, "raw");

run("Convert to Mask", "only");

run("Dilate"); // a dilation to counteract the reduction in EEF size by the blur

new_min_EEF_area = min_EEF_area/(pixelWidth*pixelHeight);

run("Analyze Particles...", " size=new_min_EEF_area-10000 pixel circularity=circ-1.00 include add");

close("EEF");

num_EEF = RoiManager.size;

if(num_EEF>0){

//Delete ROIs touching the boundary of the image since we cannot look at their surroundings

check_boundary_location(num_EEF);

//Adding ROIs of the center, and surroundings of potential EEFs

num_EEF = RoiManager.size;

select_band(num_EEF);

select_center(num_EEF);

//Measure EEF channel signal in, around & at the center of potential EEFs

deselect();

selectWindow(origtitle);

run("Duplicate...", "title=EEF duplicate channels=2");

roiManager("Deselect");

roiManager("Show none");

roiManager("Show all");

run("Set Measurements...", "mean standard display redirect=None decimal=0");

roiManager("Measure");

non_EEF = newArray();//to store signals that are not EEF

for(l=0; l<num_EEF; l++){

int1 = getResult("Mean", l);

int2 = getResult("Mean", l + num_EEF);

int3 = getResult("Mean", l + 2*num_EEF);

SD1 = getResult("StdDev", l);

ratio1 = int2/int1;

ratio2 = int3/int1;

//select EEFS with ratio1 > 0.3 or ratio2>2 and good SD

if( (ratio1>R1) | (ratio2>R2) | (SD1 < SD_min) | (SD1 > SD_max) ){

non_EEF = Array.concat(non_EEF, l);

}

}

//delete the ROIs around & at center of potential EEFs

make_selection(num_EEF, 3*num_EEF);

roiManager("Delete");

//Delete potential EEFs that shows signs of debris or background

delete_selection(non_EEF);

close("Results");

deselect();

close("EEF");

}

//Block 3, Filtering potential EEFs based on their location

//If we have potential EEFs, check their distance to the edge of organoid

num_potential_EEF = roiManager("size"); //Number of potential EEFs based on intensity

if(num_potential_EEF > 0){

selectWindow("Edge");

Create_points("Edge");//Display centers of EEFs

run("Nearest Edge");//Calculate their distance to the edge

non_EEF = newArray();

for(Row=0; Row<(nResults); Row++){

distance = getResult("dist_1", Row) * pixelWidth;

if(distance > Max_distance_to_edge){non_EEF = Array.concat(non_EEF,Row);}

}

delete_selection(non_EEF);

}

close("Results");

num_potential_EEF = roiManager("size"); //Number of potential EEFs based on intensity

if(num_potential_EEF == 0){EEF_present = false;}

else{EEF_present = true;}

//Adding centers of nuclei to ROI manager

selectWindow("Nuclei_Centers");

run("Find Maxima...", "prominence=1 output=[Point Selection]");

roiManager("Add");

//If EEFs are left after the first distance filter, check their distance to the closest nuclei

if(EEF_present == true){

smallest_dist = newArray(num_potential_EEF);

non_EEF = newArray();

for(EEF=0; EEF<num_potential_EEF; EEF++){

newImage("EEF_distance", "8-bit black", width, height, 1);

roiManager("Select", EEF);//Select an EEF and imprint its edge

run("Draw", "slice");

roiManager("Select", num_potential_EEF);//display all the centers of nuclei

run("Nearest Edge");

for(nuc=0; nuc<nResults; nuc++){

distance = getResult("dist_1", nuc);

if(nuc == 0){smallest_dist[EEF] = distance;}

if(distance < smallest_dist[EEF]){smallest_dist[EEF] = distance;}

}

smallest_dist[EEF] = smallest_dist[EEF] * pixelWidth;

close("EEF_distance");

close("Results");

if(smallest_dist[EEF] > Max_distance_to_nuclei){non_EEF = Array.concat(non_EEF,EEF);}

}

non_EEF = Array.concat(non_EEF,num_potential_EEF);//Add the center of nuclei to the selection of ROI that will be removed

delete_selection(non_EEF);//Delete all centers of nuclei and far EEFs from the ROI manager

num_potential_EEF = roiManager("size");

}

diameter = "";

maturation_signal = "";

if(EEF_present == true){

//If there are EEFs that passed the filters, calculate their largest diameter

if(num_potential_EEF > 0){

selectWindow("Maturation");

run("Set Measurements...", "mean redirect=None decimal=0");

roiManager("Measure");

for(R=0; R<nResults; R++){

maturation_signal2 = getResult("Mean", R);

maturation_signal = maturation_signal + " " + maturation_signal2/maturation_signal1;

}

close("Results");

selectWindow(origtitle);

run("Set Measurements...", "centroid fit display redirect=None decimal=0");

roiManager("Measure");

for(Row=0; Row<nResults; Row++){

diameter = diameter + " " + (getResult("Major", Row));

roiManager("Select", Row);

run("Add Selection...");

}

close("Results");

num_EEF = roiManager("Count");

}

else{num_EEF = 0;}

}

else{num_EEF = 0;}

if(num_EEF > 0){

roiManager("Deselect");

roiManager("Delete");

}

close("Results");

close("Edge");

close("Nuclei_Centers");

selectWindow(origtitle);

saveAs("tiff",save_loc+origtitle);

close("*");

print(origtitle);

print("Number of Nuclei = " + number_nuclei);

print("Number of EEF = " + num_EEF);

print("Diameters of EEFs = " + diameter);

print("Maturation signal = " + maturation_signal);

print("");

non_EEF = newArray();

}

//All the used functions

function create_hole(){

getPixelSize(unit, pixelWidth, pixelHeight);

run("Set Measurements...", "centroid fit display redirect=None decimal=3");

roiManager("multi-measure measure_all");

X = getResult("X", 0)/pixelWidth;

Y = getResult("Y", 0)/pixelHeight;

makeOval(X , Y , 1, 1);

close("Results");

roiManager("Add");

//Combine all parts of the organoids, form one ROI

roiManager("Select", newArray(0,1));

roiManager("XOR");

roiManager("Delete");

roiManager("Add");

}

function Create_points(name){

getPixelSize(unit, pixelWidth, pixelHeight);

run("Set Measurements...", "centroid fit display redirect=None decimal=3");

roiManager("Deselect");

roiManager("multi-measure measure_all");

selectWindow(name);

X = newArray();

Y = newArray();

for (Row = 0; Row < nResults; Row++) {

X[Row] = getResult("X", Row) / pixelWidth;

Y[Row] = getResult("Y", Row) / pixelHeight;

}

close("Results");

makeSelection("point", X, Y);

}

function remove_small_organoids(min_org_size){

num_org = roiManager("size");

num_nuclei = newArray(num_org);

close("Results");

selectWindow("Nuclei_Centers");

roiManager("Show all");

roiManager("Measure");

for(i = 0; i <num_org; i++){

area = getResult("Area", i);

mean = getResult("Mean", i);

num_nuclei[i] = (mean*area)/255;

}

close("Results");

small_organoids = newArray();

for(j=0; j<num_org; j++){

if(num_nuclei[j]<min_org_size){

small_organoids= Array.concat(small_organoids,j);

}

}

if(small_organoids.length > 0){

roiManager("Select", small_organoids);

roiManager("Delete");

}

}

function deselect(){

roiManager("Show all");

roiManager("Show none");

}

function make_selection(start, stop){

selection = newArray();

for(j=start; j<stop; j++){

selection = Array.concat(selection, j);

}

roiManager("Select", selection);

}

function delete_selection(array){

if(array.length>0){

roiManager("Select", array);

roiManager("Delete");

}

}

function check_boundary_location(num_EEF){

getDimensions(width, height, channels, slices, frames);

bad_location = newArray();

for(i=0; i<num_EEF; i++){

x = getResult("BX", i);

y = getResult("BY", i);

x2 = x + getResult("Width", i);

y2 = y + getResult("Height", i);

if((x == 0) | (y == 0) | (x2 == width) | (y2 == height)){

bad_location = Array.concat(bad_location, i);

}

}

close("Results");

delete_selection(bad_location);

}

function select_band(num_EEF){

for(j=0;j<num_EEF; j++){

roiManager("Select",j);

run("Make Band...", "band=band_size");

roiManager("Add");

}

}

function select_center(num_EEF){

getPixelSize(unit, pixelWidth, pixelHeight);

run("Set Measurements...", "centroid fit display redirect=None decimal=0");

for(k=0; k<num_EEF; k++){

roiManager("Select", k);

roiManager("Measure");

dia = getResult("Major", 0);

x = getResult("X", 0)/pixelWidth - (dia/2);

y = getResult("Y", 0)/pixelHeight - (dia/2);

makeOval(x , y , dia, dia);

roiManager("Add");

close("Results");

}

}
